# Deep Learning for Identifying Promising Drug Candidates in Drug–Phospholipid Complexes

**DOI:** 10.3390/molecules28124821

**Published:** 2023-06-16

**Authors:** Soyoung Yoo, Hanbyul Lee, Junghyun Kim

**Affiliations:** Department of Artificial Intelligence, Sejong University, Seoul 05006, Republic of Korea; yooso0731@gmail.com (S.Y.); 99dlgksquf@naver.com (H.L.)

**Keywords:** deep learning, drug discovery, drug–phospholipid complex, variational autoencoder, principal component analysis, convolutional neural network

## Abstract

Drug–phospholipid complexing is a promising formulation technology for improving the low bioavailability of active pharmaceutical ingredients (APIs). However, identifying whether phospholipid and candidate drug can form a complex through in vitro tests can be costly and time-consuming due to the physicochemical properties and experimental environment. In a previous study, the authors developed seven machine learning models to predict drug–phospholipid complex formation, and the lightGBM model demonstrated the best performance. However, the previous study was unable to sufficiently address the degradation of test performance caused by the small size of the training data with class imbalance, and it had the limitation of considering only machine learning techniques. To overcome these limitations, we propose a new deep learning-based prediction model that employs variational autoencoder (VAE) and principal component analysis (PCA) techniques to improve prediction performance. The model uses a multi-layer one-dimensional convolutional neural network (CNN) with a skip connection to effectively capture the complex relationship between drugs and lipid molecules. The computer simulation results demonstrate that our proposed model performs better than the previous model in all performance metrics.

## 1. Introduction

Drug discovery and development are widely recognized as time-consuming and expensive processes due to the various stages involved, including target identification and validation, lead discovery and optimization, as well as preclinical and clinical trials [1]. The development of new drugs often takes more than a decade and requires billions of dollars, with the costs increasing in proportion to the number of candidate drugs being considered [2]. Therefore, it is critical to identify promising candidate drugs to reduce costs by minimizing the number of necessary in vitro tests. In recent years, in silico approaches have gained popularity for identifying candidate drugs [3,4]. In particular, numerous studies have utilized machine learning (ML) and deep learning (DL) techniques to identify candidate drugs [5,6,7,8].

In new drug development, optimizing drug solubility is a significant challenge for pharmaceutical scientists due to the fact that roughly 40% of drugs have received market approval, and nearly 90% of candidate drugs suffer from poor water solubility problems [9]. Poor water solubility can decrease the bioavailability of drugs and ultimately limit their effectiveness. Therefore, many studies have been conducted to address this issue.

Phospholipid complexing is considered as one of the effective solutions for overcoming the poor solubility of certain drugs [10,11,12]. Phospholipids are amphipathic molecules that consist of both a polar and a non-polar portion. They can create complexes with potential drugs, such as active pharmaceutical ingredients (APIs), in aprotic solvents through various interactions. The phospholipid complex can enhance the oil solubility of hydrophilic drugs and the water solubility of hydrophobic drugs. This helps the drug enter the bloodstream after oral administration, leading to improved bioavailability.

The formation of a drug–phospholipid complex may depend on the complex’s physicochemical properties and the experimental conditions. Therefore, identifying these complexes through in vitro tests can be time-consuming and costly. These cost issues can be addressed by predicting which candidate drugs are capable of forming phospholipid complexes using in silico methods, and then conducting experiments accordingly.

In a previous study [13], the authors proposed a light gradient-boosting machine (lightGBM) model to predict the formulation of a drug–phospholipid complex. They evaluated the performance of seven different machine learning approaches: random forest (RF), support vector machine (SVM), decision tree (DT), naïve Bayes, extreme gradient boosting (XGBoost), and lightGBM. Among these approaches, lightGBM achieved the highest accuracy with a score of 87%. Furthermore, the authors carried out berberine (BBR)–phospholipid complexation experiments to identify key factors influencing the complexation rate. The experimental results aligned with the predictions made by the lightGBM model. The study’s findings suggest that machine learning models hold promise in identifying drug candidates. However, in the previous study, they did not consider any deep learning approaches, and it is noteworthy that the lightGBM model failed to achieve sufficient test performance due to the overfitting problem. Therefore, to overcome the limitations of previous research, we propose a novel DL-based model for predicting drug–phospholipid complex formulations.

The proposed model consists of a data preprocessing module for effective model training and a prediction block. The data preprocessing module includes normalization process, data augmentation process using variational autoencoder (VAE) [14], and dimensionality reduction process using principal component analysis (PCA) [15]. For the prediction block, we designed an advanced prediction model by incorporating a skip connection into a multi-layer, one-dimensional convolutional neural network (1D-CNN) that has recently achieved high performance even with its simple structure. Finally, through computer simulation, we confirmed the performance improvement of the proposed DL-based model compared to the existing lightGBM. Additionally, we evaluated the impact of the VAE technique proposed for data augmentation and various resampling techniques on the predictive performance of the model through performance comparisons.

## 2. Results and Discussion

### 2.1. Computer Simulation Results

All feature values in the original training dataset were scaled to values between 0 and 1 using min–max normalization. The class imbalance issue was resolved by adding data generated through the VAE to the original training dataset. The loss function used for VAE training consists of reconstruction loss and regularization loss. To balance the two losses, we set the weight multiplied by the reconstruction loss to 1000. We optimized the VAE using the Adam algorithm with a learning rate of 0.001 and trained for 5000 iterations.

**Figure 1:**
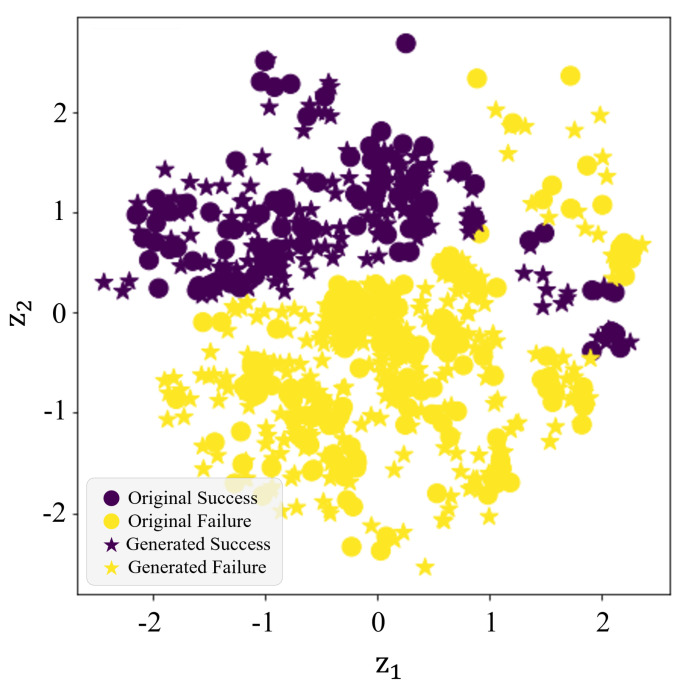
Two–dimensional (2D) latent space visualization of original and generated data samples.

We generated 500 data samples using the trained VAE. To verify the quality of the newly generated data, we visualized the class distribution of the original data samples and generated data samples in the latent space in Figure 1. In Figure 1, the $z_{1}$-axis represents the first axis of the latent space vector, and the $z_{2}$-axis represents the second axis. Circular points represent original training data, while star points represent generated data. Purple points indicate cases where drug–phospholipid complexes were formed, and yellow points indicate cases where they were not. As the class boundaries in Figure 1 are relatively distinct and data from the same class are densely clustered, we can confirm that the proposed VAE has been well trained. We also determined that the generated data has a similar distribution to the original data since the generated data is well situated in the densely clustered space by class. Therefore, we randomly selected 86 samples from the newly generated 500 data samples without replacement and added them to the original dataset to address the data imbalance.

**Figure 2:**
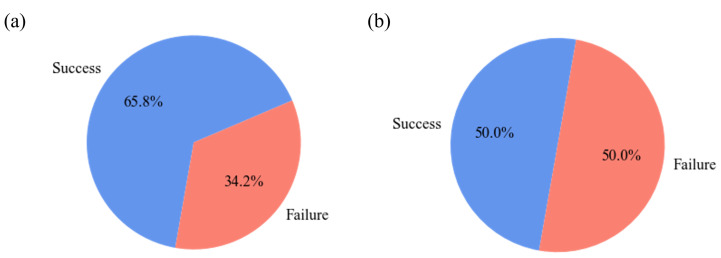
The class distribution in (**a**) the original training dataset; (**b**) the augmented training dataset.

Figure 2 illustrates the class balance of the dataset used for model training. Figure 2a represents the original training dataset’s class distribution, and Figure 2b shows the class distribution of the new training dataset balanced through data augmentation. Through this, we can confirm that the class imbalance issue in the dataset has been resolved.

**Figure 3:**
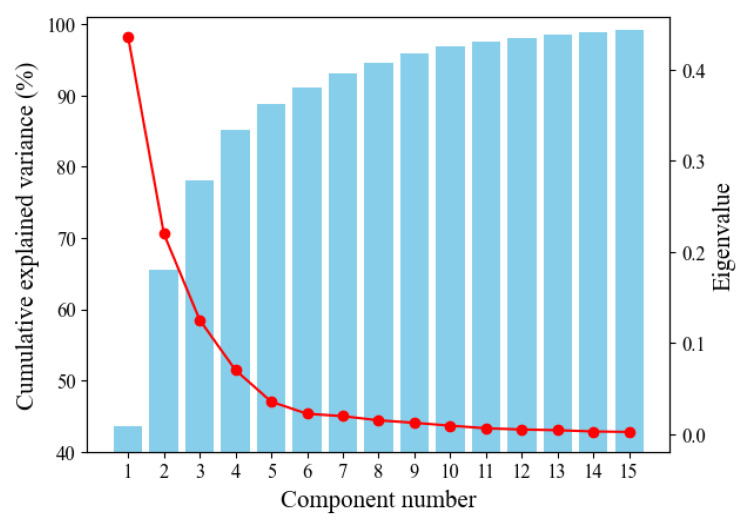
Scree plot for PCA.

As the final step in the preprocessing process, we use PCA to reduce the dimensionality of the dataset. This can also be applied identically to the test data in the test process. Figure 3 is a scree plot showing eigenvalues and cumulative variance ratios according to the component number. The blue boxes represent the cumulative variance ratio according to the principal components, corresponding to the left *y*-axis, and typically, components with cumulative values of 85% or higher are selected. The red line represents eigenvalues according to the principal component number, corresponding to the right *y*-axis, and the number of components is chosen near the part where the slope becomes significantly gentler, i.e., the “elbow”. Through the scree plot, we finally selected 10 principal components based on performance validation. As a result, we reduced the dimensionality of the training data from 46 to 10 using PCA.

The multi-layer 1D-CNN model of the drug–phospholipid complex prediction block was optimized using the Adam algorithm with a binary cross-entropy (BCE) loss function and a learning rate of 0.001. After the training for 2500 iterations using the modified training dataset, the performance on the training dataset was as follows: accuracy of 0.975, AUC of 0.998, specificity of 0.961, sensitivity of 0.989, and F1-score of 0.975. On the test dataset, the performance was: accuracy of 0.942, AUC of 0.960, specificity of 0.897, sensitivity of 0.975, and F1-score of 0.951.

In summary, the proposed model exhibited a performance of over 94% in almost all metrics for both training and test datasets. Specifically, for the test dataset, the accuracy was around 94%, and the sensitivity was 97.5%, showcasing very high performance. Furthermore, the number of learning parameters for the prediction model was 3105, and the training process took 30.18 s.

### 2.2. Performance Comparison of Data Augmentation Techniques

Data augmentation is used to address the class imbalance issue in a given original dataset [16]. In this paper, VAE was employed for data augmentation. In traditional data augmentation approaches that do not utilize DL, resampling techniques are a prominent example. Resampling techniques can be divided into oversampling, which generates additional data samples; undersampling, which removes data samples; and combined sampling, which incorporates both oversampling and undersampling methods.

We applied various resampling techniques instead of VAE to verify the prediction performance. For oversampling, we selected SMOTE [17]; for undersampling, we chose ENN [18]; and for combined sampling, we opted for SMOTE-ENN [19]. We maintained the same structure for the proposed model, with the exception of the data augmentation part.

Table 1 shows the number of class-specific data samples generated using each data augmentation technique. The number of training samples was 358 when using the oversampling method SMOTE and VAE, and 193 when using the undersampling method ENN. Each dataset was dimensionally reduced to 10 using PCA before being used for the model training.

**Figure 4:**
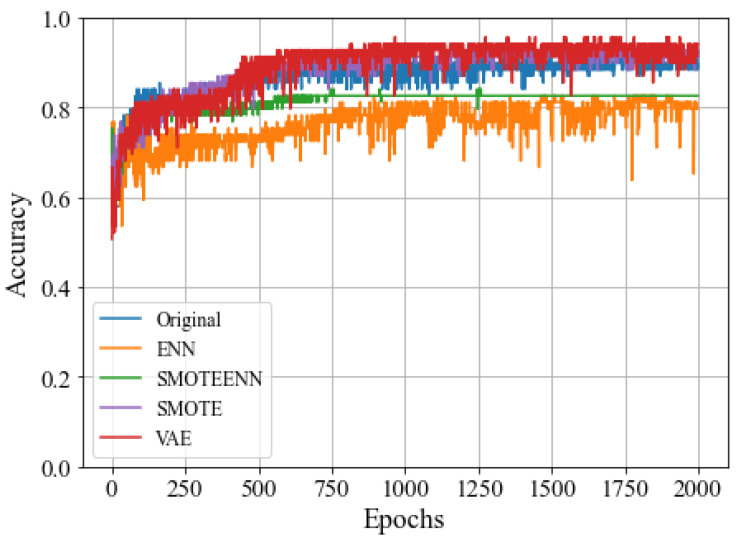
Testing accuracy comparison of sampling techniques.

Figure 4 shows the test accuracy of the resampling techniques and the proposed VAE as the number of epochs increases. The prediction accuracy of ENN and SMOTE-ENN was noticeably lower compared to other methods, and particularly worse than when using the original training dataset. The accuracy of the original dataset, SMOTE, and VAE increased similarly as the training progressed, with VAE achieving the highest performance from approximately the 1600th iteration.

Table 2 shows the prediction performance based on different data augmentation techniques. Although ENN and SMOTE-ENN, which include undersampling, demonstrated high performance in the training dataset, they overfit and resulted in a 20% performance degradation in the test dataset. Oversampling techniques such as SMOTE and VAE did not overfit and maintained high prediction performance in the test set. In comparison to the original dataset, both ENN and SMOTE-ENN experienced approximately a 10% performance drop in the test dataset, while SMOTE and VAE showed performance improvement. Notably, when using the proposed VAE, prediction accuracy improved by about 3%.

### 2.3. Performance Comparison between the Previous Model and the Proposed Model

In the prior research [13], the authors used seven machine learning models to predict the formulation of phospholipid complex and proposed lightGBM as the model with the best performance. To overcome the performance limitation of the existing model, we incorporated a data preprocessing module that assists with feature extraction in our proposed model and designed the prediction block as a multi-layer 1D-CNN with a single skip connection. We analyzed the performance of the existing model and the proposed model using the same dataset.

Table 3 shows the performance of the proposed model compared to the existing model. The proposed model performs better than the prior model in all metrics except for the F1-score on the training dataset, and particularly demonstrated an improvement of at least 5% across all metrics on the test dataset. While the accuracy gap between the training and test datasets was approximately 10% for the lightGBM model, our model achieved a gap of only 3%, indicating improved generalization performance. Additionally, the sensitivity metric, which we aimed to highlight among several performance indicators, was very high with 98.9% on the training dataset and 97.5% on the test dataset. It can be confirmed that the proposed model can identify the possibility of the phospholipid complex formation with higher performance compared to the existing model, using appropriate data preprocessing and effective prediction model construction.

### 2.4. Ablation Study

Furthermore, we conducted computer simulations for ablation study to validate the effectiveness of our proposed model. Specifically, we individually excluded VAE and PCA from our proposed model and evaluated the performance of predicting drug–phospholipid complex formation prediction. Table 4 shows the results of the simulations. In the first ablation case, where VAE was excluded, the accuracy of the training dataset was 97.1%, which was similar to the proposed model, but the accuracy of the test dataset was 91.3%, about 3% lower than the proposed model. In the second ablation case, excluding PCA, the accuracy of the training and test datasets was 90.5% and 88.4%, respectively, which was lower than the proposed model. It is noteworthy that in both ablation cases, the proposed model performs better than the previous model with a test dataset accuracy of 87%.

Thus, we were able to confirm that both VAE and PCA contribute positively to the performance of the drug–phospholipid complex formation prediction. Particularly, PCA had a greater impact on performance improvement during the training and testing processes compared to VAE, as it generates new variables through the novel combinations of various molecular descriptors. These results indicate that our proposed model is appropriately designed for predicting drug–phospholipid complex formation.

### 2.5. Analysis of Contributing Factors

We conducted an analysis of the impact of the molecular descriptors in the dataset on the prediction outcomes. We performed predictions in our proposed model excluding PCA and investigated the predictive contributions of each descriptor. Since PCA transforms the dimensions of the data, it is difficult to interpret it in terms of the original features. Therefore, we excluded PCA from our analysis.

**Figure 5:**
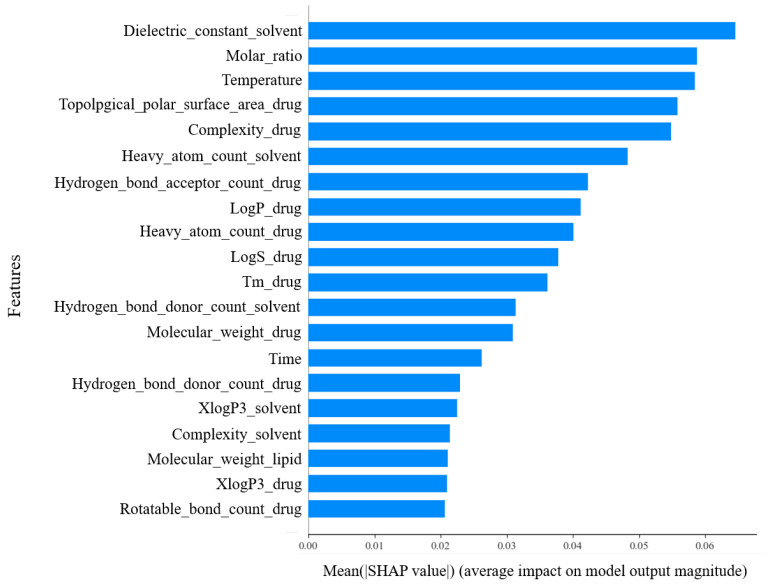
The relative importance of the molecular descriptors of the proposed model.

Figure 5 represents the 20 descriptors with relatively high contributions to the prediction of drug–phospholipid complex formation among the 46 descriptors. When compared to the results of the lightGBM model in [13], it is observed that 17 out of the top 20 descriptors match, excluding 3 descriptors. However, there are differences in the order and contribution ratios of each descriptor due to differences in the prediction models.

**Figure 6:**
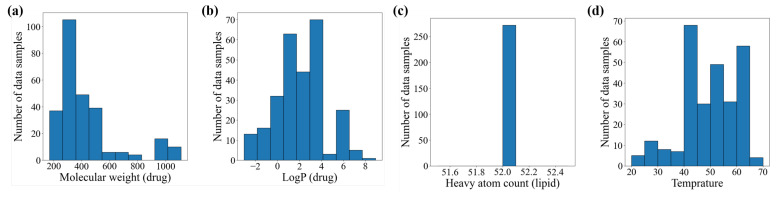
The distribution of the parameter in the original training dataset: (**a**) molecular weight for drugs; (**b**) LogP for drugs; (**c**) Heavy atom count for lipid; (**d**) Reaction temperature.

The fact that three descriptors of experimental conditions are positioned in the top ranks suggests that experimental conditions play a significant role in drug–phospholipid complex formation. Additionally, it is evident that the distribution of data significantly influences the prediction performance. For instance, the number of heavy atoms in lipids depicted in Figure 6c has only one value of 52 for all samples, regardless of the formation of drug–phospholipid complexes. Such features provide no assistance in the prediction. The reason why most molecular descriptors for the phospholipids have lower contributions is also due to this factor. Therefore, when performing predictions using such a dataset, feature selection or dimensionality reduction techniques like PCA are essential. The significant impact of PCA on performance observed in the ablation study can also be interpreted from this perspective.

On the other hand, among the molecular descriptors for the solvents, the dielectric constant exhibited the highest contribution in the proposed model, unlike the lightGBM model. This confirms the proposed neural network architecture and data augmentation have contributed to the high importance of this molecular descriptor. From the result, we can conclude that we can overcome the limitations of the given experimental data by leveraging various deep learning techniques. Additionally, the interpretable results provided by deep learning technology offer valuable insights for conducting additional experiments aimed at improving performance.

## 3. Materials and Methods

### 3.1. Materials

We utilized the drug–phospholipid complex data selected in the previous research [13]. The data were collected from published literature between 1999 and 2019 in the Web of Science, Scopus, and CNKI databases using a keyword search strategy. To ensure data reliability, the authors selected literature containing complexation rate information or other characterizations. As a result, 341 valid formulations and 59 APIs were gathered from the literature data.

The data were characterized by molecular descriptors to describe the structural and physicochemical properties of the APIs, phospholipids, and all reaction solvents. Specifically, to describe the structural and physiochemical properties of APIs, we selected 11 molecular descriptors, including molecular weight, XlogP3, hydrogen bond donor count, hydrogen bond acceptor count, rotatable bond count, topological polar surface area, heavy atom count, complexity, logP, logS, and melting temperature. Additionally, for phospholipids description, we utilized 18 molecular descriptors such as unsaturation index, hydrophilic factor, Ghose–Crippen molar refractivity, topological polar surface area, number of nitrogen atoms, etc. All reaction solvents were characterized using 13 molecular descriptors, including molecular weight, XlogP3, logP, hydrogen bond donor count, hydrogen bond acceptor count, rotatable bond count, topological polar surface area, heavy atom count, complexity, electric constant, boiling point, vapor pressure, and polar index. Furthermore, temperature and time were chosen as descriptors to explain the experimental conditions.

The entire dataset was divided into two subsets: a training set (272 formulas) and a test set (69 compositions) using the molecular distance-based feature importance sampling (MD-FIS) algorithm [20]. Since the size of the entire dataset was not large, a validation set was not defined. For each data sample, if the complexation rate was 80% or higher, it was labeled as “success”, and if it was lower than 80%, it was labeled as “failure”.

### 3.2. Proposed Model

**Figure 7:**
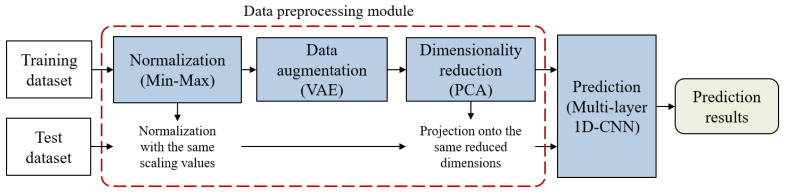
Architecture of the proposed model for phospholipid complex formation prediction.

The proposed model for predicting drug–phospholipid complex formation consists of a data preprocessing module and a prediction block. In the data preprocessing module, we employ min–max normalization for data normalization, VAE for data augmentation, and PCA for dimensionality reduction. Data augmentation is applied only to the training dataset to address the class imbalance problem. The transformed training dataset, obtained through data preprocessing, is subsequently used for training the prediction model. Within the prediction block, the model employs a multi-layer 1D-CNN structure which includes a single skip connection. The overall architecture of the proposed model is illustrated in Figure 7.

### 3.3. Min–Max Normalization

Deep learning involves the process of updating the weights and biases of neurons to minimize the loss function. When a dataset containing variables with different scales is used for deep learning training, each variable is affected differently by weight updates, which can make parameter optimization challenging and lead to a decline in prediction performance. Therefore, data standardization or normalization is employed for scaling purposes. Among them, min–max normalization is one of the most common methods. Min–max normalization is a scaling approach that utilizes the maximum and minimum values of each variable and is defined as follows:

(1) $$X_{normalization}=\frac{X-X_{min}}{X_{max}-X_{min}},$$

where *X* represents the variable to be scaled. After scaling, the variable has values ranging between 0 and 1.

To determine the necessity of the normalization technique introduced above for the dataset, we visualized the distribution of some variables within the original training dataset in Figure 6. Figure 6a–d show the distributions of molecular weight, logP, number of heavy atoms in lipids, and reaction temperature, respectively. The molecular weight and logP of the drugs ranged from 200 to 1100 and −2 to 8, as shown in Figure 6a,b. This means that the original dataset exhibits significant scale differences between variables. Since the PCA, which we will use later, can be significantly impacted by large-scale variables when selecting principal components, it is essential to standardize or normalize the data in advance. Therefore, we first applied min–max normalization, and as a result, all elements of the training dataset have values between 0 and 1.

### 3.4. Variational Autoencoder

VAE is a type of generative model that learns to represent complex data distributions by encoding input data into a lower-dimensional latent space and then decoding it back to the original space. VAE consists of two main components: an encoder and a decoder. The encoder learns to map the input data to a lower-dimensional latent space, represented by a probability distribution over latent variables. The decoder then takes samples from this distribution and reconstructs the input data.

One of the key features of VAE is the incorporation of a regularization term in their training objective. This term encourages the learned latent space to have a specific structure, typically a multivariate Gaussian distribution with a mean of zero and an identity covariance matrix. This constraint ensures that the latent space is smooth and well structured, allowing for more meaningful interpolations and sampling.

The training process of VAE involves optimizing both the encoder and decoder networks using a combination of two loss functions: the reconstruction loss and the regularization loss. The reconstruction loss measures how well the decoder can reconstruct the input data from the encoded latent variables, while the regularization loss enforces the desired structure on the latent space. By minimizing the combined loss, VAE learns to generate samples that closely resemble the training data while maintaining a well-structured latent space. Our training dataset comprises a total of 272 rows, with 179 cases involving the formation of drug–phospholipid complexes and 93 cases without drug–phospholipid complex formation, displaying imbalanced characteristics. Small and imbalanced datasets can negatively impact the generalization performance and accuracy of deep learning models in classification problems. This is because predictive models tend to be more biased towards the features of the larger class compared to the smaller class. Among various methods to address data imbalance issues, we employed a data augmentation technique called VAE. Specifically, we used VAE to generate additional data samples from the smaller class, equal to the difference in sample size between the larger and smaller classes, and incorporated them into the original dataset.

**Figure 8:**
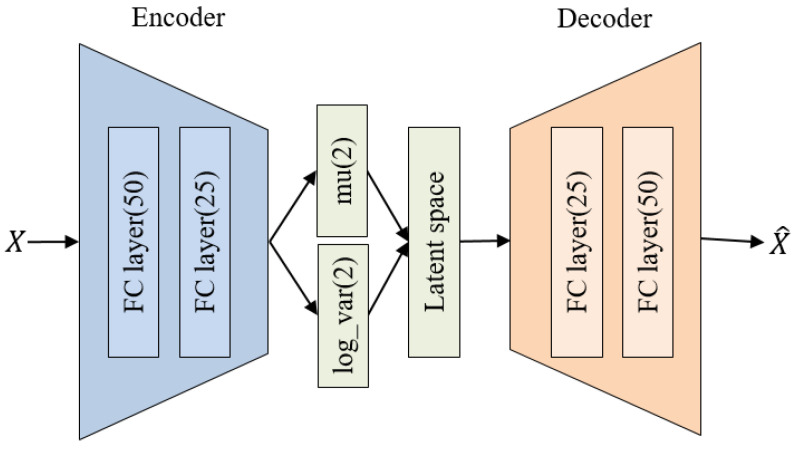
Architecture of the proposed VAE.

Figure 8 illustrates the architecture of the VAE model we constructed. All layers of the VAE are composed of fully connected layers. The encoder passes through two hidden layers with 50 and 25 neurons, respectively, and outputs the mean and log-variance for generating the latent space. The proposed VAE’s latent space is set to 2 dimensions. The decoder is symmetric to the encoder and passes through two hidden layers with 25 and 50 neurons, respectively, reconstructing the input data with the same dimensions. For activation functions, we used ReLU functions [21] in all hidden layers and a sigmoid function in the output layer. Biases in all layers were initialized to 0, and weights were initialized with random values following a He normal distribution [22].

### 3.5. Principal Component Analysis

Some features in the dataset used for model training may have little or no impact on the prediction results, be less important, or exhibit high correlations with other features. In such cases, performing dimensionality reduction before training can help reduce the model’s complexity and training time while improving prediction performance [23,24,25,26]. In the proposed model, we used PCA as a dimensionality reduction technique. PCA is a widely used statistical technique for dimensionality reduction and feature extraction in data analysis. The primary goal of PCA is to transform a dataset with multiple variables into a new set of variables called principal components while retaining as much of the original variation as possible. These principal components are linear combinations of the original variables and are orthogonal to each other, meaning that they are uncorrelated.

PCA works by calculating the covariance matrix of the original dataset, then finding the eigenvectors and eigenvalues of this matrix. The eigenvectors represent the directions of the principal components, while the eigenvalues represent the magnitude of the variance explained by each principal component. The principal components are then sorted in descending order according to their corresponding eigenvalues, with the first principal component explaining the largest amount of variance in the data, the second principal component explaining the next largest amount, and so on. By projecting the original data onto a lower-dimensional subspace formed by the top k principal components, PCA can reduce the dimensionality of the data while retaining most of the original variation. This reduced-dimensional representation can help in visualizing high-dimensional data, removing noise, and improving the performance of machine learning algorithms by reducing the risk of overfitting and the computational cost associated with high-dimensional data.

Determining the number of principal components in PCA is crucial, as it can influence the final prediction performance. To address this, we employed a scree plot. The plot shows the eigenvalues, which represent the amount of variance explained by each principal component, in descending order. Each eigenvalue is plotted on the y-axis, while the corresponding principal component’s order is plotted on the x-axis. Typically, the plot shows a steep drop in the eigenvalues initially, followed by a leveling off or an “elbow” shape. This elbow point indicates that the addition of more principal components beyond that point would not significantly improve the explained variance. To determine the optimal number of principal components, one can look for the “elbow” or the point where the plot starts to level off. Retaining principal components up to this point ensures that a substantial amount of the original variance is preserved while reducing the dimensionality of the data. Alternatively, by calculating the cumulative explained variance, the components that achieve the cumulative value of 85% or higher can be chosen as the principal components.

### 3.6. Multi-Layer 1D-CNN

A 1D-CNN [27] is a variation of the traditional CNN designed to handle one-dimensional data, such as time series or sequences. Like regular CNNs, 1D-CNNs consist of several layers, including convolutional layers, pooling layers, and fully connected layers. The main difference is that the convolution and pooling operations are performed only along one spatial dimension instead of two, as in the case of 2D-CNNs for images.

In a 1D-CNN, the convolutional layer employs one-dimensional filters that move across the input sequence, identifying local patterns or characteristics within a specific window or receptive area. The learned filters can capture temporal dependencies or other local structures within the data. After the convolutional layers, pooling layers are often used to reduce the dimensionality of the feature maps and extract the most important features. Commonly used pooling techniques in 1D-CNNs include max pooling, average pooling, and global average pooling. Finally, the output from the convolutional and pooling layers is typically flattened and passed through one or more fully connected layers to produce the final output, such as a classification or regression result. In recent studies, such 1D-CNN models have achieved high performance with only fewer than 10,000 parameters [28,29,30].

**Figure 9:**
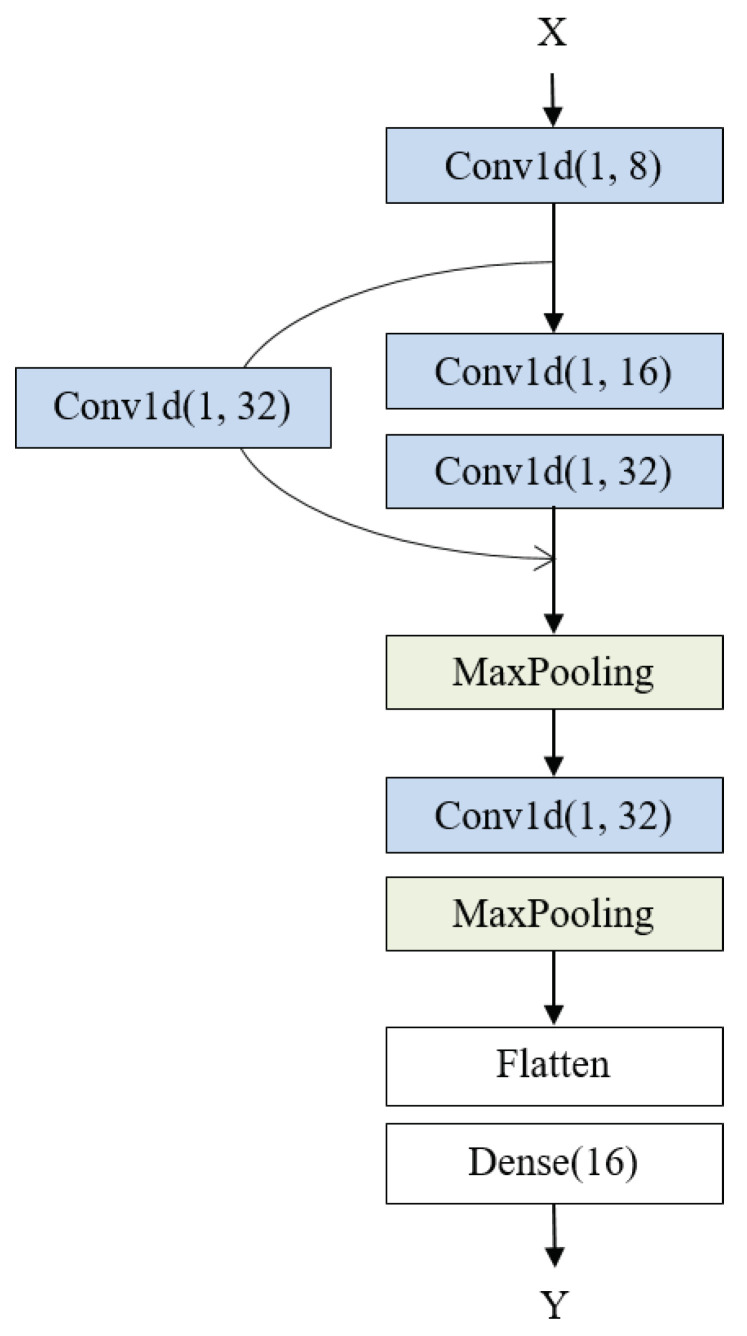
Architecture of the proposed multi-layer 1D-CNN.

Figure 9 illustrates the proposed multi-layer 1D-CNN architecture. We configured the kernel size and stride of the 1D-CNN to be 1 and increased the number of channels sequentially as the layers deepen to 8, 16, and 32. Here, the number of channels refers to the number of filters for feature extraction, and increasing this number allows for more complex and diverse feature extraction.

The feature maps generated by the first 1D convolutional layer are combined with the feature maps generated by the third 1D convolutional layer to prevent the loss of information from the lower layers. Since increasing the number of channels could lead to overfitting, we mitigated this risk by adding a pooling layer. In the proposed architecture, the pooling size is set to 2. After the pooling, additional convolution and pooling layers are applied to extract the final features. The final output feature maps are flattened through a flatten layer and then passed through a dense layer to produce the predicted values for the drug–phospholipid complex formation.

### 3.7. Evaluation Metrics

For comparison with previous research [13], the same metrics were used for performance evaluation. Accuracy is commonly used to evaluate model performance in classification tasks. It is the ratio of correct predictions to total predictions made. It is defined as follows:

(2) $$Accuracy=\frac{TP+TN}{TP+FP+FN+TN},$$

where *TP* = true positive, *FP* = false positive, *TN* = true negative, and *FN* = false negative.

From the perspective of predicting drug–phospholipid complex formation, *TP* represents the number of cases where the model accurately predicts the formation of a complex when it is indeed formed. *FP* represents the number of cases where the model erroneously predicts the formation of a complex when it is not actually formed. *TN* represents the number of cases where the model correctly predicts that no complex is formed when it is indeed not formed. FN represents the number of cases where the model incorrectly predicts that a complex is not formed when it is actually formed.

Sensitivity, also known as recall, is the ratio of correctly predicted positive samples to the total actual positive samples. It is defined as follows:

(3) $$Sensitivity=\frac{TP}{TP+FN}.$$

Specificity is the ratio of correctly predicted negative samples to the total actual negative samples. It is defined as follows:

(4) $$Specificity=\frac{TN}{TN+FP}.$$

Precision is the ratio of correctly predicted positive samples to the total predicted positive samples. It is defined as follows:

(5) $$Precision=\frac{TP}{TP+FP}.$$

*F*1-score is the harmonic mean of precision and recall, often used for binary classification problems with imbalanced classes. It is defined as follows:

(6) $$F1=\frac{2\times (Precision\times Recall)}{(Precision+Recall}$$

Area under the curve (AUC) is a performance metric that is often used in binary classification problems to evaluate the effectiveness of a model. Specifically, it measures the area under the receiver operating characteristic (ROC) curve, which is a plot that illustrates the true positive rate, sensitivity against the false positive rate, and 1-specificity at various classification threshold levels.

The AUC value ranges from 0 to 1, with an AUC of 0.5 representing a random classifier and an AUC of 1 representing a perfect classifier. In general, the higher the AUC value, the better the classification model’s performance. AUC is particularly useful for evaluating classification models when the classes are imbalanced or when there is a varying cost associated with false positives and false negatives. Additionally, AUC takes both sensitivity and specificity into account, providing a comprehensive view of the classifier’s performance.

All of the performance metrics mentioned above have values ranging from 0 to 1, with higher values indicating better predictive performance of the model. The predictive model serves as a preliminary screening tool to identify as many candidate drugs as possible that may form drug–phospholipid complexes. Therefore, in this study, it is particularly important for the prediction model to maximize sensitivity, along with accuracy and other performance metrics.

## 4. Conclusions

In this paper, we addressed a drug–phospholipid complex formation prediction technique that can be utilized to identify promising candidate complexes prior to experimental validation. In previous studies, we compared the performance of seven machine learning models for predicting drug–phospholipid complex formation and suggested lightGBM as the best-performing model. However, the existing model had limitations in demonstrating sufficiently high performance on the test dataset. Therefore, we propose a new prediction model based on deep learning to prevent overfitting and ensure high generalization performance. In detail, we devised a preprocessing module to tackle the challenges of scale disparities in variables and class imbalance in the original training dataset. This module incorporates min–max normalization, data augmentation using VAE, and dimensionality reduction via PCA. Furthermore, to facilitate efficient prediction model design, we utilized a multi-layered 1D-CNN with skip connections.

From the computer simulation results, it is confirmed that the proposed VAE yielded the most significant improvement in prediction performance when compared to various resampling techniques. Additionally, comparing the proposed model with the existing model using lightGBM, it was evident that the proposed model exhibited superior prediction performance even without the proposed data preprocessing steps. The final proposed model, incorporating the preprocessing steps, effectively reduced the accuracy discrepancy between the training and test datasets approximately from 10% to 3% and achieved an improvement in test dataset accuracy from 87% to 94.2%. Furthermore, the proposed model successfully identified promising candidate drugs with a high sensitivity of 97.5%. These results provide valuable insights into the necessity of the preprocessing module to address class imbalance issues in the dataset and enable the development of efficient prediction models. Taking into account the overall results of this study, it is expected that the proposed model and techniques will significantly contribute to diverse potential applications in the process of identifying candidate compounds for new drug development.

## Figures and Tables

**Table 1:** Class distribution comparison between original and new training datasets.

	Variable		Original	ENN	SMOTE	SMOTE-ENN	VAE
Drug–phospholipid complexation		Success	179	100	179	138	179
		Failure	93	93	179	137	179
	Total		272	193	358	275	358

**Table 2:** Performance comparison of the sampling techniques for the proposed model.

Methods	Training Set					Test Set				
	Accuracy	AUC	Specificity	Sensitivity	F1-Score	Accuracy	AUC	Specificity	Sensitivity	F1-Score
Original	0.967	0.997	0.946	0.978	0.975	0.913	0.936	0.862	0.95	0.927
ENN	0.985	0.999	0.978	0.99	0.985	0.797	0.846	0.897	0.725	0.806
SMOTE-ENN	1	1	1	1	1	0.826	0.828	0.793	0.85	0.85
SMOTE	0.966	0.998	0.978	0.955	0.966	0.928	0.957	0.931	0.925	0.937
VAE	0.975	0.998	0.961	0.989	0.975	0.942	0.960	0.897	0.975	0.951

**Table 3:** Performance comparison between the existing and the proposed models.

Model	Training Set					Test Set				
	Accuracy	AUC	Specificity	Sensitivity	F1-Score	Accuracy	AUC	Specificity	Sensitivity	F1-Score
Gao et al. [13]	0.971	0.998	0.946	0.983	0.978	0.870	0.906	0.793	0.925	0.892
Prop.	0.975	0.998	0.961	0.989	0.975	0.942	0.960	0.897	0.975	0.951

**Table 4:** Computer simulation results for the ablation study.

Model	Training Set					Test Set				
	Accuracy	AUC	Specificity	Sensitivity	F1-Score	Accuracy	AUC	Specificity	Sensitivity	F1-Score
Without VAE	0.971	0.998	0.946	0.983	0.978	0.913	0.928	0.828	0.975	0.929
Without PCA	0.905	0.980	0.855	0.955	0.910	0.884	0.902	0.793	0.950	0.905
Prop.	0.975	0.998	0.961	0.989	0.975	0.942	0.960	0.897	0.975	0.951

## Data Availability

The datasets used in this study are available at https://www.sciencedirect.com/science/article/pii/S0009261420302694#m0005 (accessed on 15 May 2023).
